# Age- and sex-specific reference values of biventricular flow components and kinetic energy by 4D flow cardiovascular magnetic resonance in healthy subjects

**DOI:** 10.1186/s12968-023-00960-x

**Published:** 2023-09-18

**Authors:** Xiaodan Zhao, Ru-San Tan, Pankaj Garg, Ping Chai, Shuang Leng, Jennifer Ann Bryant, Lynette L. S. Teo, Tee Joo Yeo, Marielle V. Fortier, Ting Ting Low, Ching Ching Ong, Shuo Zhang, Rob J. Van der Geest, John C. Allen, Teng Hong Tan, James W. Yip, Ju Le Tan, Marina Hughes, Sven Plein, Jos J. M. Westenberg, Liang Zhong

**Affiliations:** 1 National Heart Research Institute Singapore, National Heart Centre Singapore, Singapore, Singapore; 2 Duke-NUS Medical School, Singapore, Singapore; 3 Department of Cardiovascular Medicine, University of East Anglia, Norwich, UK; 4 National University Hospital Singapore, Singapore, Singapore; 5 Yong Loo Lin School of Medicine, National University of Singapore, Singapore, Singapore; 6 KK Women’s and Children’s Hospital, Singapore, Singapore; 7 Singapore Institute for Clinical Sciences, A*STAR, Singapore, Singapore; 8 Philips Healthcare Germany, Hamburg, Germany; 9 Department of Radiology, Leiden University Medical Center, Leiden, The Netherlands; 10 Leeds Institute of Cardiovascular and Metabolic Medicine, University of Leeds, Leeds, UK; 11 National Heart Centre Singapore, Singapore, Singapore

**Keywords:** 4D flow, Kinetic energy, Flow components, Hemodynamics, Reference values

## Abstract

**Background:**

Advances in four-dimensional flow cardiovascular magnetic resonance (4D flow CMR) have allowed quantification of left ventricular (LV) and right ventricular (RV) blood flow. We aimed to (1) investigate age and sex differences of 4D flow CMR-derived LV and RV relative flow components and kinetic energy (KE) parameters indexed to end-diastolic volume (KEi_EDV_) in healthy subjects; and (2) assess the effects of age and sex on these parameters.

**Methods:**

We performed 4D flow analysis in 163 healthy participants (42% female; mean age 43 ± 13 years) of a prospective registry study (NCT03217240) who were free of cardiovascular diseases. Relative flow components (direct flow, retained inflow, delayed ejection flow, residual volume) and multiple phasic KEi_EDV_ (global, peak systolic, average systolic, average diastolic, peak E-wave, peak A-wave) for both LV and RV were analysed.

**Results:**

Compared with men, women had lower median LV and RV residual volume, and LV peak and average systolic KEi_EDV_, and higher median values of RV direct flow, RV global KEi_EDV_, RV average diastolic KEi_EDV_, and RV peak E-wave KEi_EDV_. ANOVA analysis found there were no differences in flow components, peak and average systolic, average diastolic and global KEi_EDV_ for both LV and RV across age groups. Peak A-wave KEi_EDV_ increased significantly (r = 0.458 for LV and 0.341 for RV), whereas peak E-wave KEi_EDV_ (r = − 0.355 for LV and − 0.318 for RV), and KEi_EDV_ E/A ratio (r = − 0.475 for LV and − 0.504 for RV) decreased significantly, with age.

**Conclusion:**

These data using state-of-the-art 4D flow CMR show that biventricular flow components and kinetic energy parameters vary significantly by age and sex. Age and sex trends should be considered in the interpretation of quantitative measures of biventricular flow.

*Clinical trial registration* https://www.clinicaltrials.gov. Unique identifier: NCT03217240.

**Supplementary Information:**

The online version contains supplementary material available at 10.1186/s12968-023-00960-x.

## Introduction

Accurate assessment of intracardiac blood flow is important in the assessment and clinical management of various cardiovascular diseases [1, 2]. Two-dimensional (2D) phase-contrast cardiovascular magnetic resonance (CMR) flow is commonly used in clinical practice to quantify conventional flow parameters such as mean and peak velocities and stroke volume. However, 2D imaging with one-directional velocity encoding is unable to capture complex multi-directional blood flow patterns inside the heart and great vessels. Four-dimensional (4D) CMR flow imaging enables acquisition of comprehensive blood flow in three spatial directions simultaneously within a volume of interest resolved over time, and can provide new hemodynamic parameters such as flow components and kinetic energy (KE) beyond the conventional flow parameters. The clinical application of 4D flow CMR and the diagnostic potential of derived parameters for systolic and diastolic assessment have been comprehensively reviewed [1–3].

Kinetic energy is an important part of the external work of the heart that is performed to accelerate blood from the resting state to the current velocity. Research interest in left ventricular (LV) and right ventricular (RV) KE has burgeoned [4–15]. RV KE has been shown to be significantly increased in repaired tetralogy of Fallot (rTOF) [6–8] and decreased in pulmonary arterial hypertension (PAH) [10] compared with controls. Additionally, LV direct-flow average KE was shown to be a risk prognosticator in heart failure [11]. In contrast, RV flow components are rarely assessed, except in small cohorts of healthy subjects [4, 16], rTOF [8] and PAH [10, 17], where healthy subjects have been used as controls. Direct flow, which describes blood that enters and exits the ventricle in the analysed cardiac cycle, has been observed contributing to a larger portion of the end-diastolic volume (EDV) in RV than LV, and is of high importance when assessing RV diastolic function [18]. RV direct flow has been shown to be independently associated with RV dysfunction, adverse RV remodeling and exertional capacity in rTOF [8] and PAH [10], moreover, it had better discrimination than RV ejection fraction in terms of area under curve for adverse RV remodeling and intermediate and high risk exercise capacity [8, 10]. Therefore, a standardized set of 4D flow CMR-derived parameters, with well-defined references ranges will be necessary in order to better understand and quantify RV hemodynamic changes in various pathological states. The impact of age and sex on 4D flow CMR-derived LV flow components and KE has been investigated [12, 13] and so far, only one paper has specifically reported age-associated effects on RV KE parameters among healthy subjects (n = 53) [14]. To our knowledge, no study has examined the age- and sex associated changes on RV flow components nor specifically focused on the sex trends on RV kinetic energy in a large healthy cohort. Therefore, the current study aims to fill these knowledge gaps by investigating age- and sex trends of 4D flow CMR-derived biventricular relative flow components and KE parameters indexed to end-diastolic volume (KEi_EDV_) in a large cohort of healthy subjects.

## Methods

### Study population

From June 2017 to February 2022, 185 healthy subjects aged 20–80 years were identified from a prospective study, which was a multicenter registry of healthy volunteers and patients with congenital heart disease or pulmonary hypertension (*ClinicalTrials.gov* identifier NCT03217240). The exclusion criteria for healthy subjects were history of: (1) non-cardiac illness with a life expectancy of less than 2 years; (2) previous heart, kidney, liver or lung transplantation; (3) history of any major medical problems, cardiovascular disease or cardiovascular risk factor (e.g., hypertension, diabetes or dyslipidemia) or significant renal or lung disease; (4) taking medications for cardiovascular disease or cardiovascular risk factor (e.g., for hypertension) and (5) smoking (defined as over 5 sticks per day or who had quit smoking for less than 12 months and had smoked over 5 sticks per day previously). Part of the study population was included in our previous publications to investigate the impact of age, sex and ethnicity on LV flow components and KE [12], and associations of 4D flow components and KE parameters with RV functional, remodeling and cardiopulmonary exercise testing (CPET) outcomes in rTOF patients [8] and PAH patients [10]. After excluding subjects with no/incomplete CMR scan (n = 6), CMR scans without/incomplete 4D flow (n = 12) and inadequate image quality for 4D flow analysis (n = 4), 163 healthy subjects were included in the final analysis. All subjects were stratified by age into five groups: 20–29 years (n = 31, M/F: 18/13), 30–39 years (n = 45, M/F: 25/20), 40–49 years (n = 38, M/F: 23/15), 50–59 years (n = 29, M/F: 14/15) and 60–70 years (n = 20, M/F: 15/5). This study had been approved by the Institutional Review Boards, and written informed consent was obtained from each subject.

### Cardiac magnetic resonance protocol

CMR acquisition was performed on 3.0 T Ingenia (Philips Healthcare, Best, The Netherlands) and 1.5 T Magnetom Aera (Siemens Healthineers, Erlangen, Germany) scanners, as previously published [8, 10, 12]. Balanced steady-state free precession end-expiratory breath-hold cine images were acquired for the 2-, 3- and 4-chamber long-axis and a stack of short-axis images covering the entire LV and RV, and reconstructed with a temporal resolution of 30 frames per heart cycle. Whole heart 4D flow CMR was acquired per guideline recommendations [19, 20]. Typical cine and 4D flow CMR acquisition parameters in different centers are provided in Additional file 1: Table S1.

### Cardiac magnetic resonance image analysis

All CMR image analysis were performed at a core laboratory using commercial research software MASS (Version 2019EXP, Leiden University Medical Center, Leiden, The Netherlands).

#### Biventricular measurements

A semi-automated method based on artificial intelligence (AI) and subsequent manual inspection and corrections where needed was used to segment endocardial and epicardial borders in stacks of LV and RV short-axis images [21]. In our study cohort, manual adjustments of AI segmented contours in heart apex were performed in 32/163 (19.6%) cases. Papillary and trabecular muscles were included in the volume calculation. End-diastolic volume (EDV) and end-systolic volume (ESV) were defined respectively as maximal and minimal values of the volume curve. LV mass was estimated at end-diastole. LV mass and all volumetric parameters were indexed to body surface area (BSA).

#### 4D flow error corrections

Correction for local velocity offset errors was applied prior to particle tracing. First, for images acquired on the Philips scanner, concomitant gradient correction and local phase offset correction was performed as provided by the scanner software. Second, for images acquired on the Siemens scanner, local velocity offset correction using a 2nd order static tissue plane fit method was performed in MASS. Third, the residual velocity offset errors for both scanners were further minimized by subtracting the median velocity within the LV myocardial region at the end-systolic (ES) moment for all voxels and at every time phase, in accordance with the study by Kamphuis et al. [22].

#### Biventricular 4D flow analysis

The analysis techniques and definitions of both LV and RV four flow components (direct flow, retained inflow, delayed ejection flow, residual volume) and KE parameters normalized to EDV (global, peak systole, average systole, average diastole, peak E-wave and peak A-wave KEi_EDV_) have been described in previous publications [8, 10, 12]. Briefly, spatial misalignment between short-axis cine and 4D flow acquisitions was corrected by a rigid registration using the open-source Elastix registration toolbox [23]. The particle tracking algorithm used fourth-order Runge–Kutta numerical integration with a time-step of one fifth of the temporal resolution of the 4D flow acquisition (i.e., 8 ms). The number of particles released was dependent on the LV and RV end-diastolic volume using a particle size of 3 mm × 3 mm × 3 mm. The positions of the particles were evaluated at the previous and subsequent ES phases, and the particle tracing results were visually reviewed to confirm the absence of any remaining offset errors. Particles located within the LV (or RV) and below the LV (or RV) basal plane were included in the flow component calculations.

**Fig. 1:**
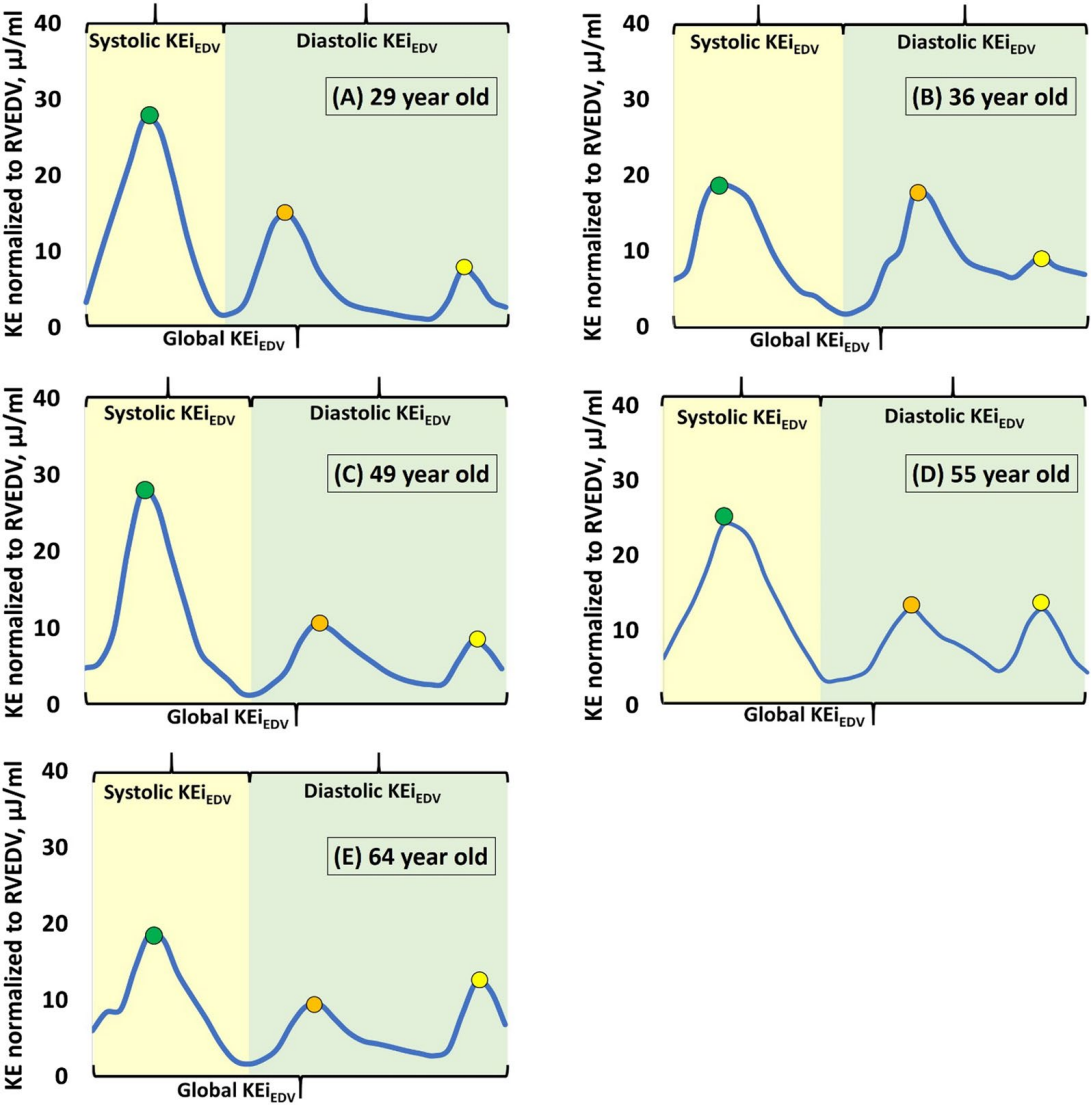
Right ventricular (RV) kinetic energy (KE) curve normalized to RV end-diastolic volume (EDV) in **A** a 29-year-old normal subject, **B** a 36-year-old normal subject, **C** a 49-year-old normal subject, **D** a 55-year-old normal subject and **E** a 64-year-old normal subject. *KEi*_*EDV*_ KE normalized to RVEDV

Phasic endocardial and epicardial contours from LV and RV were used for flow component and KE analyses. The positions of the traced particles at ES were used to classify flow into four functional components [24, 25]: (1) direct flow: blood that enters and exits the ventricle in the analyzed cardiac cycle; (2) retained inflow: enters the ventricle but does not exit during the analyzed cycle; (3) delayed ejection flow: starts within the ventricle and exits during the analyzed cycle; and (4) residual volume: blood that remained in the ventricle for the duration of at least one full cardiac cycle. Each component volume was indexed to the total ventricular EDV (LV EDV for LV flow components, and RV EDV for RV flow components). For each volumetric element (voxel), KE was computed using the following formula:

$${\text{KE }} = { 1}/{2}\cdot\rho_{{{\text{blood}}}} \cdot{\text{V}}_{{{\text{voxel}}}} \cdot{\text{ v}}_{{{\text{voxel}}}}^{2} ,$$

where ρ_blood_ represents blood density (1.06 g/cm^3^); V_voxel_, voxel volume; and v_voxel_, velocity of the corresponding voxel. All KE parameters were normalized to EDV (KEi_EDV_) and presented in μJ/ml. KEi_EDV_ parameters at physiologically relevant epochs (global [entire cycle], systole, diastole) and cardiac cycle time points (peak systole, peak E-wave, peak A-wave) were extracted from the time-resolved KEi_EDV_ curve. Good reproducibility of 4D flow CMR-derived LV and RV parameters had been demonstrated in our previous study [8, 10, 12]. Examples of RV KEi_EDV_ curve in each of the five age groups are illustrated in Fig. 1. The corresponding videos of the four RV flow components with appropriate color legends for the whole cardiac cycle are provided in Additional file 2.

### Statistical analysis

Data were analysed using SPSS (version 25.0, Chicago, IL, USA). Continuous variables were expressed as mean ± standard deviation (SD) for normally distributed data or median (25th percentile, 75th percentile) for non-normally distributed data. The mean ± SD representations for 4D flow parameters were also provided in the supplemental tables to facilitate comparisons with previous findings and also provide reference values for future relevant studies. Comparison of means between two groups was analysed using two-sample *t* test for normally distributed data; Mann–Whitney U tests for non-normally distributed data; and Kruskal–Wallis (K–W) non-parametric one-way ANOVA for more than two groups with post-hoc pair-wise comparisons in the event of a significant K-W test with Bonferroni corrections where appropriate. The Chi-square test or Fisher’s exact test, as appropriate, were used for analysis of categorical variables. Bonferroni significance levels were calculated when comparing sex and age with biventricular 4D flow CMR parameters. For other results, only raw P-values were reported. Associations between continuous variables were investigated using regression and correlation (Pearson). Statistical significance was declared at *P* < 0.05.

## Results

### Demographic characteristics and baseline CMR data

Demographics and LV and RV function measurements for all subjects and subjects stratified by sex are shown in Table 1. Among 163 subjects, the mean age was 43 ± 13 years with M/F: 95/68. Compared with men, women weighed less, were of shorter height, had lower systolic and diastolic blood pressures, BSA and body mass index; women also had smaller LV mass index, EDV index, ESV index, stroke volume (SV) index and smaller RVEDV/LVEDV ratio, and higher ejection fractions (EF) for both LV and RV (all *P* < 0.05). There were no significant differences in age and heart rate between men and women.

**Table 1:** Demographics, flow components and kinetic energy (KE) parameters for the overall population, men and women

	All (n = 163)	Men (n = 95)	Women (n = 68)	*P*^*1,2*^
Demographics				
Age, years	43 ± 13	44 ± 13	42 ± 12	0.322
Gender, M/F	95/68	95/0	0/68	–
Weight, kg	65 ± 12	71 ± 10	56 ± 9	** < 0.001**
Height, cm	167 ± 9	172 ± 7	160 ± 7	** < 0.001**
Systolic blood pressure, mmHg	125 ± 16	129 ± 15	121 ± 17	**0.002**
Diastolic blood pressure, mmHg	76 ± 12	79 ± 11	73 ± 12	**0.003**
Body surface area, m^2^	1.72 ± 0.19	1.83 ± 0.14	1.57 ± 0.14	** < 0.001**
Body mass index, kg/m^2^	23.1 ± 3.4	24.0 ± 3.4	21.8 ± 3.1	** < 0.001**
Heart rate, bpm	73 ± 13	73 ± 14	73 ± 12	0.809
Ethnicity				0.529*
Chinese, n (%)	143 (87.7%)	84 (88.4%)	59 (86.8%)	–
Malay, n (%)	7 (4.3%)	5 (5.3%)	2 (2.9%)	–
Other, n (%)	13 (8%)	6 (6.3%)	7 (10.3%)	–
LV function				
LV mass index, g/m^2^	47 ± 11	51 ± 12	42 ± 8	** < 0.001**
LVEDV index, ml/m^2^	71 ± 13	75 ± 12	65 ± 11	** < 0.001**
LVESV index, ml/m^2^	27 ± 9	29 ± 8	23 ± 8	** < 0.001**
LVSV index, ml/m^2^	44 ± 8	46 ± 7	42 ± 7	**0.001**
LV ejection fraction, %	63 ± 8	62 ± 7	65 ± 8	**0.022**
RV function				
RVEDV index, ml/m^2^	77 ± 15	83 ± 14	69 ± 13	** < 0.001**
RVESV index, ml/m^2^	34 ± 10	38 ± 10	29 ± 8	** < 0.001**
RVSV index, ml/m^2^	43 ± 8	45 ± 8	40 ± 6	** < 0.001**
RV ejection fraction, %	56 ± 7	55 ± 6	59 ± 6	** < 0.001**
RVEDV/LVEDV ratio	1.09 ± 0.13	1.11 ± 0.12	1.07 ± 0.13	**0.018**
LV flow components^a^				
Direct flow, %	34 (29, 38)	33 (28, 37)	34 (31, 40)	0.132
Retained inflow, %	15 (12, 18)	14 (12, 17)	16 (14, 19)	0.041
Delayed ejection flow, %	17 (14, 20)	16 (13, 19)	17 (15, 22)	0.092
Residual volume, %	33 (29, 37)	35 (30, 39)	31 (27, 34)	** < 0.001**
LV KEi_EDV_ parameters^a^				
Global KEi_EDV_, μJ/ml	9.1 (7.5, 11.4)	9.0 (7.5, 11.3)	9.3 (7.6, 11.5)	0.894
Peak systolic KEi_EDV_, μJ/ml	18.3 (15.1, 22.9)	20.1 (16.0, 24.3)	16.6 (13.6, 20.4)	**0.002**
Average systolic KEi_EDV_, μJ/ml	10.2 (8.1, 12.6)	10.8 (8.8, 13.4)	9.2 (7.6, 11.3)	**0.002**
Average diastolic KEi_EDV_, μJ/ml	8.7 (6.8, 10.7)	8.3 (6.3, 10.3)	9.1 (7.3, 11.5)	0.054
Peak E-wave KEi_EDV_, μJ/ml	25.2 (19.0, 31.5)	23.7 (17.8, 30.2)	27.7 (22.7, 33.0)	0.010
Peak A-wave KEi_EDV_, μJ/ml	10.8 (6.8, 15.2)	11.3 (7.1, 15.9)	10.2 (6.8, 14.3)	0.543
KEi_EDV_ E/A ratio	2.39 (1.40, 3.70)	2.10 (1.25, 3.70)	2.85 (1.65, 3.71)	0.060
RV flow components^a^				
Direct flow, %	35 (31, 40)	34 (30, 38)	38 (32, 42)	**0.007**
Retained inflow, %	17 (14, 20)	17 (14, 19)	17 (14, 20)	0.892
Delayed ejection flow, %	16 (13, 19)	15 (12, 18)	17 (13, 20)	0.043
Residual volume, %	30 (26, 37)	33 (28, 37)	27 (24, 35)	**0.001**
RV KEi_EDV_ parameters^a^				
Global KEi_EDV_, μJ/ml	8.3 (6.8, 10.3)	7.5 (6.3, 9.8)	8.9 (7.6, 10.8)	**0.005**
Peak systolic KEi_EDV_, μJ/ml	21.3 (18.0, 27.4)	21.3 (18.4, 28.7)	21.2 (17.7, 27.2)	0.718
Average systolic KEi_EDV_, μJ/ml	12.0 (9.6, 14.3)	11.9 (9.1, 14.3)	12.4 (9.7, 14.4)	0.744
Average diastolic KEi_EDV_, μJ/ml	6.1 (4.8, 7.6)	5.6 (4.3, 7.5)	6.7 (5.7, 8.4)	** < 0.001**
Peak E-wave KEi_EDV_, μJ/ml	12.9 (9.5, 16.3)	11.9 (8.5, 14.9)	14.4 (11.3, 17.9)	** < 0.001**
Peak A-wave KEi_EDV_, μJ/ml	8.3 (5.7, 11.1)	8.2 (5.9, 11.2)	8.3 (5.2, 11.4)	0.996
KEi_EDV_ E/A ratio	1.41 (0.97, 2.47)	1.30 (0.93, 2.21)	1.68 (1.15, 2.64)	0.028

Data were represented as mean ± SD or ^a^median (25th percentile, 75th percentile). *EDV* end-diastolic volume, *ESV* end-systolic volume, *KEi*_*EDV*_ KE normalized to EDV, *LV* left ventricle, *RV* right ventricle

*P value from Fisher’s exact test; ^1^*P* value from Mann–Whitney U-Test; ^2^Bonferroni significance levels for LV flow components, LV KEi_EDV_ parameters, RV flow components and RV KEi_EDV_ parameters are calculated as 0.05/4 = 0.0125; 0.05/7 = 0.007; 0.05/4 = 0.0125 and 0.05/7 = 0.007, respectively. Bold values denote statistical significance

### Reference values of LV and RV 4D flow parameters

Median values of four flow components (direct flow, retained inflow, delayed ejection flow and residual volume) were 34%, 15%, 17% and 33% for LV; and 35%, 17%, 16% and 30% for RV. Median global, average systolic and average diastolic KEi_EDV_ were 9.1 µJ/ml, 10.2 µJ/ml, 8.7 µJ/ml for LV and 8.3 µJ/ml, 12.0 µJ/ml, 6.1 µJ/ml for RV, respectively (Table 1). Median peak systolic, peak E-wave, peak A-wave, and KEi_EDV_ E/A ratio were 18.3 µJ/ml, 25.2 µJ/ml, 10.8 µJ/ml and 2.39 for LV, and 21.3 µJ/ml, 12.9 µJ/ml, 8.3 µJ/ml and 1.41 for RV, respectively (Table 1). Mean ± SD representations of LV and RV 4D flow parameters for all subjects can be found in Additional file 1: Table S2.

### Sex differences of LV and RV 4D flow parameters

**Fig. 2:**
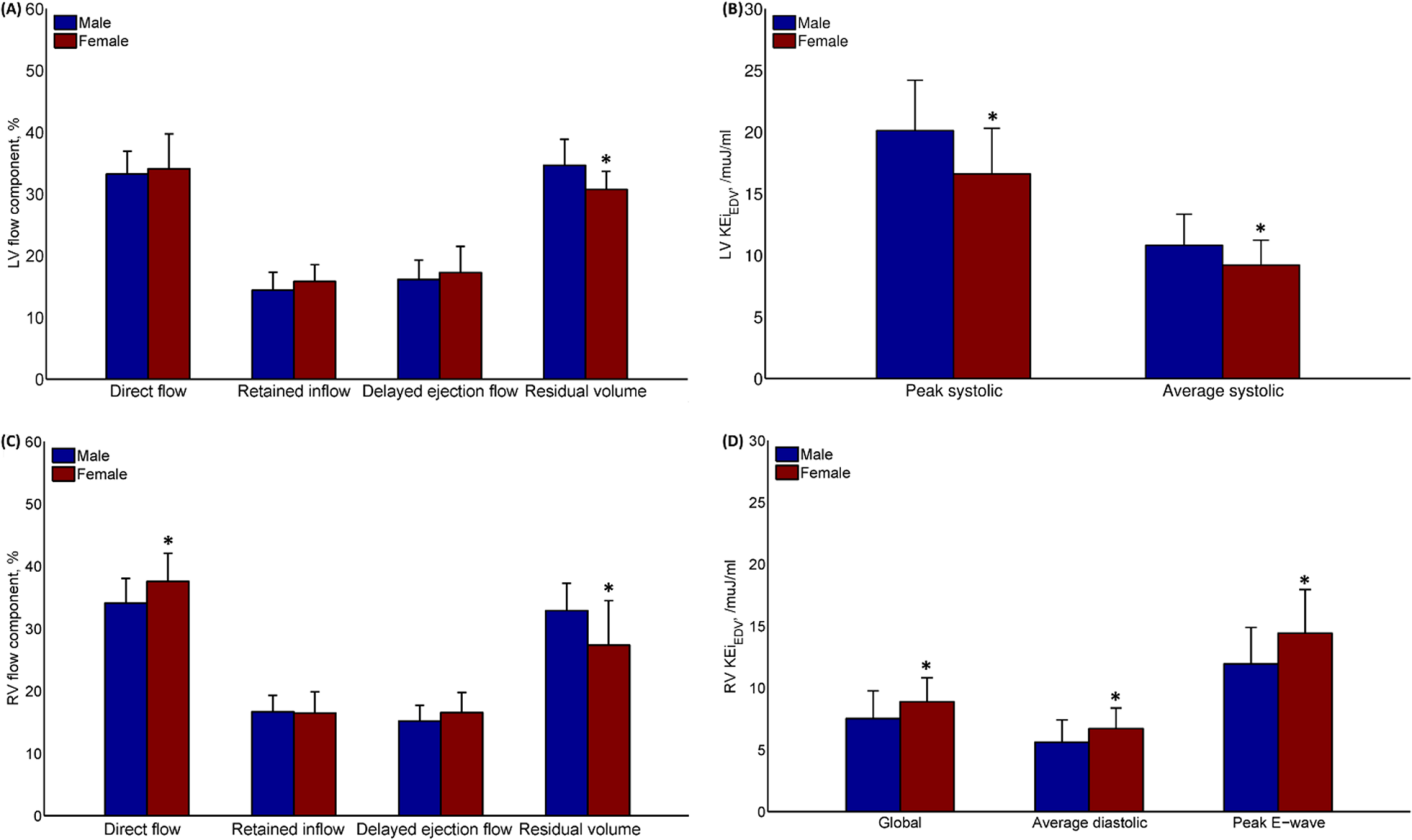
Comparisons between female and male for (**A**) left ventricular (LV) flow components; **B** LV peak systolic and average systolic KEi_EDV_; **C** right ventricular (RV) flow components; and **D** RV global, average diastolic, and peak E-wave KEi_EDV_. *KEi*_*EDV*_ Kinetic energy normalized to end-diastolic volume (EDV). *denotes *P*** < **0.0125 for (**A, C**) and *P*** < **0.007 for (**B, D**) based on Bonferroni significance levels from Table 1

Women had significantly lower LV and RV residual volume, and higher RV direct flow than men in terms of median values (all *P *< 0.0125, Table 1; Fig. 2A, C). Global and average diastolic KEi_EDV_ had no differences between the sexes for LV, while significantly increased in women for RV (both *P* < 0.007, Table 1; Fig. 2D); systolic phase (average and peak) KEi_EDV_ decreased in women for LV (both *P* < 0.007, Table 1; Fig. 2B), while no differences for RV between the sexes. RV peak E-wave KEi_EDV_ increased in women (*P* < 0.007, Fig. 2D), and peak A-wave KEi_EDV_ and E/A ratio were similar between the sexes for both LV and RV (Table 1). Mean ± SD representations of LV and RV 4D flow parameters for men and women can be found in Additional file 1: Table S2.

### Age differences of LV and RV 4D flow parameters

The median and interquartile range (IQR) values of LV and RV 4D flow parameters for each age group are given in Table 2. No significant differences were observed for four flow components, median global, peak systolic, average systolic and average diastolic KEi_EDV_ across all age groups for both LV and RV. Peak E-wave, peak A-wave and KEi_EDV_ E/A ratio had significant difference among five age groups for both LV and RV (Table 2). The 60–70 year old age group had significantly higher LV peak A-wave KEi_EDV_ and lower RV peak E-wave KEi_EDV_ compared with groups with age < 50 (both *P* ≤ 0.007). LV and RV 4D flow parameters for each age group in terms of mean ± SD can be found in Additional file 1: Table S3.

**Table 2:** 4D flow components and kinetic energy (KE) parameters according to age groups

	20–29 (n = 31)	30–39 (n = 45)	40–49 (n = 38)	50–59 (n = 29)	60–70 (n = 20)	*P*^*1*^
Gender, M/F	18/13	25/20	23/15	14/15	15/5	0.447
LV ejection fraction, %	61 ± 7	63 ± 7	64 ± 9	64 ± 8	63 ± 7	0.419
RV ejection fraction, %	55 ± 6	55 ± 7	58 ± 8	58 ± 6	56 ± 5	0.164
LV flow components^a^						
Direct flow, %	37 (33, 41)	33 (29, 36)*	33 (28, 39)*	33 (28, 39)*	33 (28, 37)	0.110
Retained inflow, %	14 (11, 17)	16 (12, 19)	16 (13, 17)	16 (14, 19)	16 (12, 19)	0.294
Delayed ejection flow, %	16 (13, 19)	17 (14, 19)	17 (14, 20)	16 (13, 22)	18 (16, 20)	0.793
Residual volume, %	33 (27, 37)	33 (30, 37)	33 (29, 38)	33 (27, 39)	32 (27, 38)	0.951
LV KEi_EDV_ parameters^a^						
Global KEi_EDV_, μJ/ml	9.5 (7.7, 11.2)	8.8 (7.0, 11.4)	8.5 (7.9, 11.0)	8.9 (7.1, 11.6)	10.2 (7.3, 12.2)	0.922
Peak systolic KEi_EDV_, μJ/ml	17.8 (14.5, 22.9)	18.5 (15.6, 25.4)	17.0 (14.3, 23.0)	18.0 (13.3, 22.0)	18.6 (14.5, 22.8)	0.951
Average systolic KEi_EDV_, μJ/ml	10.0 (8.4, 12.6)	10.1 (7.8, 13.3)	10.0 (8.3, 12.3)	9.8 (7.3, 12.6)	10.7 (9.0, 14.4)	0.815
Average diastolic KEi_EDV_, μJ/ml	8.8 (7.3, 11.3)	8.5 (6.4, 10.5)	8.2 (6.9, 10.6)	8.7 (6.2, 10.8)	9.2 (6.8, 13.1)	0.874
Peak E-wave KEi_EDV_, μJ/ml	30.9 (23.8, 37.4)	27.9 (23.9, 32.3)	23.5 (19.0, 30.1)	18.5 (14.5, 29.1)*^$^	20.1 (16.0, 24.3)*^$^	** < 0.001**
Peak A-wave KEi_EDV_, μJ/ml	9.0 (6.5, 11.4)	8.0 (5.6, 13.4)	10.6 (6.8, 15.3)	13.4 (10.3, 18.6)*^$^	18.0 (12.9, 25.3)*^$#^	** < 0.001**
KEi_EDV_ E/A ratio	3.49 (2.81, 4.63)	3.44 (2.26, 5.23)	2.10 (1.53, 3.26)*^$^	1.43 (0.90, 2.27)*^$#^	1.16 (0.81, 1.47)*^$#^	** < 0.001**
RV flow components^a^						
Direct flow, %	34 (29, 39)	35 (30, 40)	36 (31, 41)	37 (33, 40)	37 (32, 40)	0.504
Retained inflow, %	16 (14, 18)	17 (14, 18)	16 (14, 19)	17 (13, 22)	18 (15, 22)	0.625
Delayed ejection flow, %	17 (13, 19)	16 (13, 19)	16 (12, 19)	14 (11, 20)	17 (11, 19)	0.511
Residual volume, %	29 (27, 38)	33 (28, 37)	30 (25, 35)	30 (24, 38)	26 (25, 36)	0.322
RV KEi_EDV_ parameters^a^						
Global KEi_EDV_, μJ/ml	9.0 (7.0, 11.3)	8.3 (6.9, 10.2)	8.5 (7.3, 9.6)	7.7 (6.6, 10.9)	7.3 (5.6, 9.2)	0.268
Peak systolic KEi_EDV_, μJ/ml	23.1 (19.4, 30.9)	21.3 (18.3, 27.3)	21.7 (18.4, 28.0)	20.5 (16.3, 26.1)	19.3 (16.6, 27.0)	0.305
Average systolic KEi_EDV_, μJ/ml	12.7 (11.3, 16.9)	11.9 (9.7, 13.9)	12.3 (10.0, 14.4)	11.7 (8.8, 13.4)	11.0 (8.5, 12.3)	0.181
Average diastolic KEi_EDV_, μJ/ml	6.6 (5.2, 8.6)	6.0 (4.9, 7.7)	6.1 (5.3, 7.5)	6.2 (4.6, 8.3)	5.1 (3.9, 8.0)	0.467
Peak E-wave KEi_EDV_, μJ/ml	14.8 (11.6, 21.0)	13.8 (12.1, 16.5)	12.7 (9.7, 16.0)	11.3 (8.0, 14.6)	9.3 (7.0, 12.7)*^$#^	**0.001**
Peak A-wave KEi_EDV_, μJ/ml	6.9 (4.8, 8.7)	7.2 (4.5, 9.4)	8.7 (7.0, 11.2)	10.3 (8.1, 14.8)*^$^	9.7 (7.8, 12.7)*	** < 0.001**
KEi_EDV_ E/A ratio	1.95 (1.59, 3.05)	2.13 (1.36, 3.02)	1.32 (1.06, 2.15)*	0.94 (0.73, 1.35)*^$#^	1.03 (0.68, 1.25)*^$#^	** < 0.001**

Data were represented as mean ± SD or ^a^median (25th percentile, 75th percentile). *EDV* end-diastolic volume, *KEi*_*EDV*_ KE normalized to EDV, *LV* left ventricle, *RV* right ventricle

^1^*P* value from Kruskal–Wallis (K–W) non-parametric one-way ANOVA analysis. Bonferroni significance levels between any two age groups for LV flow components, LV KEi_EDV_ parameters, RV flow components and RV KEi_EDV_ parameters are calculated as 0.05/4 = 0.0125; 0.05/7 = 0.007; 0.05/4 = 0.0125 and 0.05/7 = 0.007, respectively. *Significant difference compared with 20–29 age group; ^$^significant difference compared with 30–39 age group; ^#^significant difference compared with 40–49 age group. Bold values denote statistical significance

### Association of function and 4D flow parameters with age

**Fig. 3:**
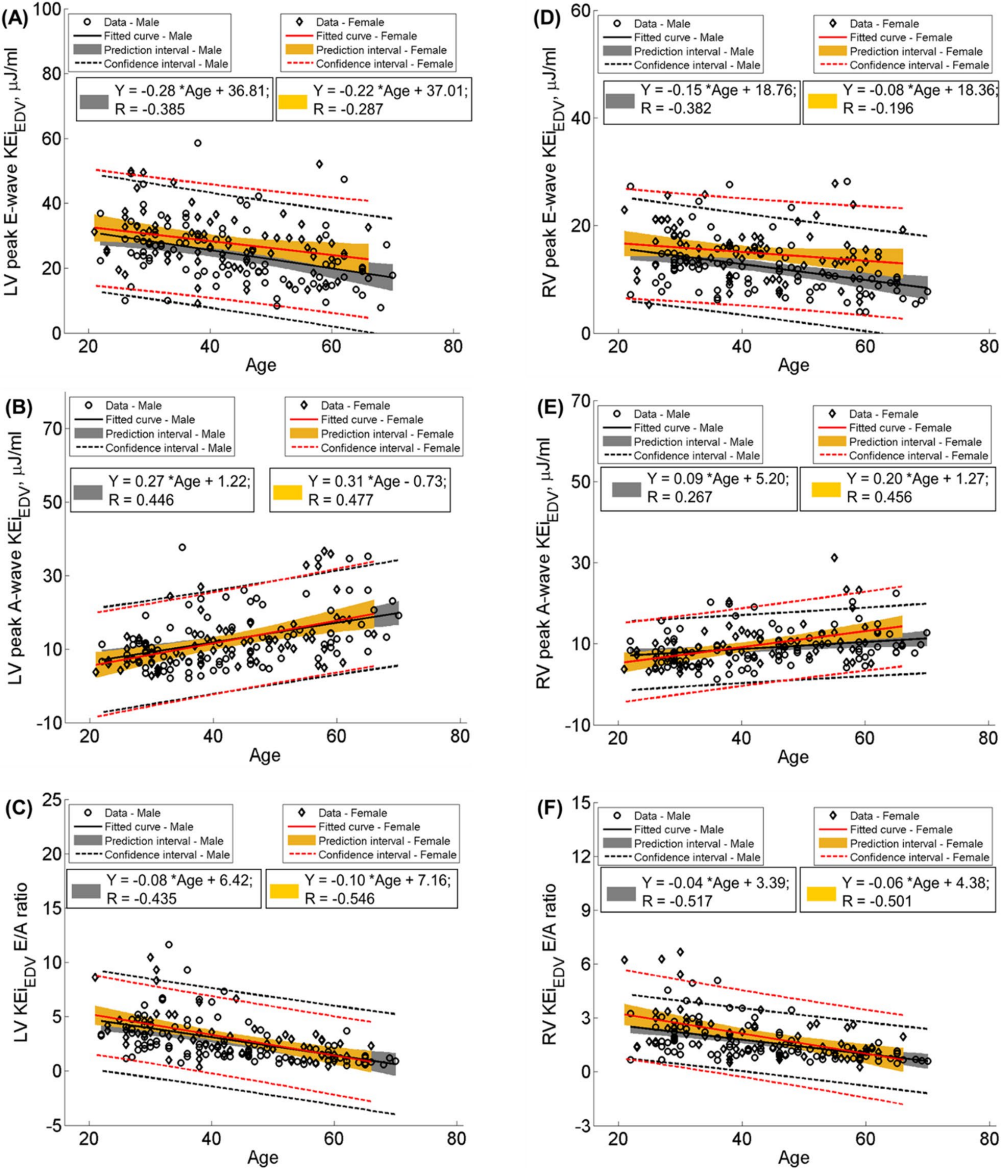
Scatter plots of peak E-wave (first row), peak A-wave KEi_EDV_ (middle row) and KEi_EDV_ E/A ratio (last row) according to age and sex for LV (left panel) and RV (right panel). All figures show regression line, correlation coefficient R, fitted curve (solid line), prediction interval (shaded area) and confidence interval (dot lines). *LV* left ventricle, *RV* right ventricle, *KEi*_*EDV*_ kinetic energy normalized to RV end-diastolic volume

Both LV and RV EDV index, ESV index and SV index were negatively associated with age (Table 3, r = − 0.270, − 0.227 and − 0.200 for LV, r = − 0.275, − 0.251 and − 0.216 for RV, respectively). Ejection fraction, flow components, global and average diastolic KEi_EDV_ were uncorrelated with age for both LV and RV (Table 3). Negative associations of RV peak systolic and average systolic KEi_EDV_ with age were observed (r = − 0.167, *P* = 0.033 and r = − 0.158, *P* = 0.044), while LV peak systolic and average systolic KEi_EDV_ were uncorrelated with age. Negative associations were found between age and peak E-wave (r = − 0.355 for LV, and r = − 0.318 for RV, *P* < 0.001) (Table 3), more so in men (r = − 0.385 for LV and r = -0.382 for RV, *P* < 0.01) (Fig. 3A, D); peak A-wave KEi_EDV_ was positively correlated with age (r = 0.458 for LV, and r = 0.341 for RV, *P* < 0.001) (Table 3), more so in women (r = 0.477 for LV and r = 0.456 for RV, *P* < 0.01) (Fig. 3B, E); KEi_EDV_ E/A ratio was negatively associated with age (r = -0.475 for LV and − 0.504 for RV, both *P* < 0.001) (Table 3), more so in women for LV (r = − 0.546, Fig. 3C) and in men for RV (r = − 0.517, Fig. 3F). Regression equations, confidence and prediction intervals are also presented in the scatter plots of Fig. 3.

**Table 3:** Correlation coefficients of 4D flow components and kinetic energy (KE) parameters with age in all subjects

	Left ventricle		Right ventricle	
	Correlation coefficient	*P* value	Correlation coefficient	*P* value
Function				
End-diastolic volume index	− 0.270	**0.001**	− 0.275	** < 0.001**
End-systolic volume index	− 0.227	**0.004**	− 0.251	** < 0.001**
Stroke volume index	− 0.200	**0.011**	− 0.216	**0.006**
Ejection fraction	0.099	0.210	0.132	0.094
4D flow components				
Direct flow	− 0.117	0.139	0.131	0.094
Retained inflow	0.064	0.415	0.109	0.165
Delayed ejection flow	0.080	0.310	− 0.153	0.051
Residual volume	0.014	0.862	− 0.118	0.134
4D KEi_EDV_ parameters				
Global KEi_EDV_	0.062	0.435	− 0.095	0.228
Peak systolic KEi_EDV_	0.036	0.649	− 0.167	**0.033**
Average systolic KEi_EDV_	0.106	0.176	− 0.158	**0.044**
Average diastolic KEi_EDV_	0.022	0.785	− 0.042	0.592
Peak E-wave KEi_EDV_	− 0.355	** < 0.001**	− 0.318	** < 0.001**
Peak A-wave KEi_EDV_	0.458	** < 0.001**	0.341	** < 0.001**
KEi_EDV_ E/A ratio	− 0.475	** < 0.001**	− 0.504	** < 0.001**

*EDV* end-diastolic volume, *KEi*_*EDV*_ KE normalized to EDV. Bold values denote statistical significance

## Discussion

To the best of our knowledge, this is the largest study to investigate age- and sex differences for 4D flow CMR derived LV and RV flow components and KE indexed to end-diastolic volume. We found that (1) women had higher RV direct flow, RV global, RV average diastolic, and RV peak E-wave, and lower LV and RV residual volume, LV peak systolic and average systolic KEi_EDV_ compared to men; (2) for both LV and RV, ageing was associated with decrease in early diastolic KEi_EDV_ and increase in late diastolic KEi_EDV_, but not with global and average diastolic KEi_EDV_ or relative flow components.

### Reference values of LV and RV 4D flow CMR parameters

With advances of 4D flow CMR techniques and availability of analysis tools, there have been multiple studies investigating 4D flow CMR-derived flow components and KE parameters, but most of the studies have focused on the LV. Our previous study established normal values of LV flow components and KE (n = 74, 34 female) [12], and the mean values in the current study were in agreement with those reported in [12] for LV flow components and KEi_EDV_ parameters but with a larger number of healthy subjects (Additional file 1: Table S2). As in previous 4D flow CMR studies [8, 10, 16, 17], RV flow components were categorized into 4 components with the same definitions. However, different software analyses might result in slight discrepancies in the values. Direct flow refers to the blood that enters the ventricle during diastole and leaves the ventricle during systole, and the median values were comparable between RV and LV in our study. Retained inflow and delayed ejection flow should be proportionately equivalent in healthy subjects, which was observed in our study (mean values: 17 vs. 17%, Additional file 1: Table S2). Fredriksson et al. [4] assessed the RV flow components in 10 healthy subjects (mean age: 46 ± 11 years, M/F: 6/4), and observed different mean values of direct flow (44 vs. 35%) and residual volume (23 vs. 31%) compared with our results, but similar mean retained inflow (17 vs. 17%) and delayed ejection flow (15 vs. 17%). Another study by Fredriksson et al. [16] calculated the RV flow components in 11 healthy controls (mean age: 67 ± 4 years, M/F: 2/9) using EnSight, and reported similar RV direct flow (44%) and residual volume (24%), but different retained inflow (19%) and delayed ejection flow (13%). Wang et al. [17] studied 14 healthy controls (mean age: 44 ± 12 years, M/F: 5/9) using CVI^42^, and reported slightly different values compared to our results: direct flow (40.7 vs. 35%), retained inflow (19.1 vs. 17%), delayed ejection flow (19.5 vs. 17%) and residual volume (20.3 vs. 31%). All these discrepancies might be explained by differences in sample sizes, which were smaller in the aforementioned studies. Moreover, age- and sex-associated changes were not analysed in the above studies. Of note, our current findings are in line with our recent publication [8, 10] but with a larger sample size.

Non-indexed RV KE parameters were reported for 9 healthy volunteers (mean age: 39 ± 15 years, M/F: 6/3) using Ensight [6]; and RV KE, both unindexed and indexed to stroke volume parameters, were calculated for 14 healthy volunteers (mean age: 30 ± 7 years, M/F: 12/2) using Segment with an in-house algorithm [7]. We reported RV flow components and KE indexed to RVEDV in our previous studies with 49 healthy subjects [8] and 51 healthy subjects [10], all of whom were included in the current analysis. The above studies all used healthy subjects as control group, and none of these studies investigated the impact of age and sex on the reported parameters. Until now, only Barker et al. [14] has published on the normal ranges of RV KEi_EDV_ parameters in 53 healthy volunteers between the ages of 20–80 years old (mean age: 45 ± 17 years, M/F: 32/21), most of whom were Caucasian. Their reported mean values were smaller compared to our results for RV global (4.6 vs. 8.8 µJ/ml), average systolic (8.12 vs. 12.3 µJ/ml), average diastolic (2.68 vs. 6.6 µJ/ml), peak E-wave (5.53 vs. 13.4 µJ/ml), peak A-wave (4.59 vs. 9.2 µJ/ml) and KEi_EDV_ E/A ratio (1.51 vs. 1.80), respectively (Additional file 1: Table S2). By the KE definition, only blood accelerated into the ventricle will cause an increase in KE. As discussed by Carlsson et al. [5], the more the valve slides over the blood volume, the less is the need for acceleration of blood through suction, and hence less KE is generated. We postulate that these discrepant findings could be explained by the relatively small sample size, larger RVEDV and longitudinal displacements observed in Caucasians compared with Asians [26].

### Association of sex with LV and RV 4D flow parameters

It is well known that women have smaller heart size and larger ejection fraction than men, and sex differences in cardiac function have been reported in congenital heart disease [27]. As stated in [28], better understanding of the sex differences is urgently needed, and sex-specific diagnostic criteria should be used when diagnosing cardiac disease in women. The sex differences in LV 4D flow components have been previously discussed in 74 healthy subjects [12]. In addition, Rutkowski et al. [15] reported LV KE differences between 20 male and 19 female healthy subjects with mean ages 26 and 27 years, respectively. Our findings expanded on these by including older subjects, reporting additional LV/RV flow components and RV KE parameters, and analysed the age trend of these parameters. As they only provided non-indexed LV KE values, direct comparison could not be made. However, as previously discussed [12], the LV peak systolic KE in both sexes were higher than our prior findings in [12], with lower values in women for both studies. Direct flow and delayed ejection flow collectively constitute the ejection portion of RV volume, which was significantly higher in women (55 vs. 49%, *P* < 0.001). Increase in RV direct flow in women could be due to the higher RVEF, as they were positively correlated (r = 0.228, *P* = 0.003). Similarly, higher systolic performance in women based on RV global longitudinal strain (GLS) and RA reservoir strain (peak strain during systole) measurements had been reported in Asians [29]. There were no significant sex differences in RV average and peak systolic KEi_EDV_, and similar findings were observed for peak tricuspid annular (TA) systolic velocity and TA displacement at end-systole [29]. Unlike Barker et al. [14], we observed significantly larger RV global, average diastolic, and peak E-wave KEi_EDV_ in women compared to men. Leng et al. [29] also observed that women had significantly higher RV early diastolic GLS rate and RA conduit strain rate (peak strain rate during early diastole) compared with men using CMR feature tracking. The higher RV peak E-wave KEi_EDV_ in women was possibly due to the smaller RVEDV as non-indexed RV peak E-waves were comparable between women and men (1.65 vs. 1.87 mJ, *P* = 0.082). As discussed by Carlsson et al. [5], RV early diastolic filling is attributable to a high degree to the return of the atrioventricular plane toward the base of the heart. TA early diastolic velocities were similar in both sexes [29], which could explain the lack of significant sex differences in non-indexed peak E-wave KE values. Comparable values of RV peak A-wave KEi_EDV_ in both sexes imply that after taking account of RVEDV, the amount of blood accelerated into the RV in late diastole is similar.

### Association of age with LV and RV 4D flow parameters

In our study, we observed that the biventricular EDV index, ESV index and SV index had significant negative correlations with age (all *P* < 0.01), which is in line with previous literature [30], and corroborates similar observations in healthy Singaporean Chinese [31] and the MESA-right ventricle study [32]. Relative proportions of the four LV and RV flow components were preserved across all age groups, implying a lack of association with age, which is consistent with the finding between age and LV flow components [12]. Similarly, RV global and average diastolic KEi_EDV_ were comparable across all age groups and were not associated with age, which are consistent with previous findings [14]. RV peak systolic and average systolic KEi_EDV_ had significant associations with age (*P* = 0.033 and *P* = 0.044), which had not been not observed in Barker et al. [14], possibly due to the small sample size in the latter. Indeed, findings of RV peak E-wave and KEi_EDV_ E/A ratio decreasing, and peak A-wave KEi_EDV_ increasing, with age have also been observed in other populations [14]. Innelli et al. [33] found a progressive reduction of early diastolic peak velocity and increase of late diastolic peak velocity on measurement of lateral tricuspid annular (TA) velocity using pulsed tissue Doppler in 298 healthy subjects (mean age: 42 ± 18 years, M/F: 186/112). Leng et al. [29] also observed similar age associations of RV velocities, RV global longitudinal strain rates, and right atrial (RA) strain rates during diastole derived from semi-automatic feature tracking in the four-chamber view in 360 healthy subjects (mean age: 50 ± 17 years, M/F: 180/180).

### Influence of vendors and acceleration methods on flow component analysis

In this study, two different vendors and field strengths (3.0 T Philips and 1.5 T Siemens) were used for CMR image acquisition, with protocols for 4D flow CMR based on the latest consensus recommendations [19]. 4D flow CMR using echo-planar imaging (EPI) had good in-scan consistency and strong scan-rescan reproducibility for the LV inflow and outflow assessment [23]. As discussed by Westenberg et al. [34], only the flow velocity in the non-blip EPI direction is correctly encoded, and in particular (high) velocities in the direction of the readout gradient are fraught with systematic inaccuracies. These inaccuracies seem to be within reasonable error limits for valvular flow evaluations [34]. Flow component analysis has been demonstrated feasible and applicable for different sequences and different scanners [8–10, 16, 17]. However, the differences in valid particle tracing and four flow components between different accelerations (EPI versus non-EPI) are unknown, and we speculate that flow components are not so much sensitive to EPI and non-EPI as they are calculated basing on full cardiac cycle particle tracing within the full cardiac cavity. In age- and sex-matched subgroups from Philips (EPI) and Siemens (non-EPI), we found they had comparable numbers of valid particles (90 ± 4% vs. 91 ± 8%, *P* = 0.530, Additional file 1: Table S4) and nonsignificant RV flow components. The results in current study indicated that vendors/scan accelerations did not influence the flow component analysis in healthy subjects. Additionally, we found that the retained inflow, delayed ejection flow and their differences were comparable between scanners and field strength in healthy subjects (Additional file 1: Table S4).

### Influence of phase offset correction on flow component and kinetic energy analysis

While correction for phase offset errors is recommended in consensus guidelines on 4D flow CMR applications [19], the magnitude and impact of uncorrected phase offset errors on KE measurements have not been studied. We performed preliminary analysis in 10 Philips data and 10 Siemens data to assess the impact of phase offset error corrections on biventricular 4D flow component and KE. For Philips data, as part of the phase offset errors were already corrected in the scanner, only subtraction of median velocity within the myocardial region at ES was not applied in Mass. For Siemens data, 2nd order static tissue plane fit method and subtraction of median velocity within the myocardial region at ES were not applied in Mass. Using nonparametric Wilcoxon Signed-Ranks Test, we found that all KEi_EDV_ parameters for both LV and RV had no significant differences for both Philips and Siemens data (Additional file 1: Table S5), and only LV direct flow had significant difference for Philips data (*P* = 0.011, Additional file 1: Table S5). Therefore, a small offset (in the order of 1 cm/s) will have little impact on KE quantification since KE of the complete ventricle is mainly determined by the velocity of in- and outflow blood having a velocity in the range of 50–80 cm/s. In contrast, for particle tracing, an error of 1 cm/s is significant since such an error will result in particles drifting away over a distance of 1 cm/s within one cardiac cycle (assuming a heart rate of 60 bpm). The comprehensive investigation of the impact of phase offset error correction is warranted in future research.

### Clinical perspective

Biventricular blood flow components and energetics derived from contemporaneous 4D flow CMR measurements offer novel insights into intracavity flow and hemodynamic changes. Reduced RV direct flow was observed in rTOF [8], PAH [10] and pediatric Fontan patients [35]. In our prior publications, we found RV direct flow to be independently associated with RV dysfunction, adverse RV remodeling and impaired exercise capacity in rTOF [8] and PAH [10]. In heart failure patients, Stoll et al. have shown that LV direct-flow average KE, but not LV ejection fraction or volumes, was an independent predictor of 6-min walk test [11]. KE computed by summing the KE of each voxel, may provide more comprehensive clinical information than 2D measurements. Therefore, further investigations are needed to obtain a deeper understanding of the physiological effects of age and gender differences on intracardiac flow parameters. The current study takes a first step in addressing the age- and sex differences and trends for LV and RV 4D flow parameters, and reporting the associations of age and sex with these, in a sizable cohort.

### Study limitations

There were some limitations to this study. First, the sample size in the 60–70 years age group is relatively small (n = 20, 5 females) as it is challenging to recruit older subjects free of cardiovascular diseases. In addition, the size for each age group is unequal and the gender in each group is imbalanced. However, age- and gender trends of the 4D flow parameters can still be observed. Future studies with adequate and balanced numbers in the various age groups stratified by sex are needed to establish age- and sex-based normal references. Second, 4D flow CMR images were acquired using two different scanners (Philips and Siemens), which is a common practice in multicenter studies. We mitigated potential differences by standardizing the acquisition procedures as much as possible to be consistent with consensus recommendations [19, 20]. Thirdly, unlike the Philips scanner, prospective electrocardiogram (ECG) triggering was used in the Siemens scanner. To ensure the diastolic phase was fully covered and the late diastolic peak flow not missed, we deliberately set the RR interval to exceed the actual RR interval, resulting longer scan times than retrospective ECG triggering. Fourthly, in the current study, we normalized KE to end-diastolic volume, which was adopted in previous studies [8–10, 12–14]. However, a few studies have normalized KE to stroke volume [7, 15], body surface area [6, 7] and cardiac output [7]. Therefore, when referencing the flow components and KE results in the current study, special attention should be paid to the normalization parameter. Lastly, our healthy subjects were identified based on their past medical history. High readings of blood pressure measurements may appear at the time of CMR, we did not disqualify the subjects from participation in the study so long as the subjects did not have a past history of hypertension. White coat hypertension is not uncommon in our experience, especially among older adults [36]. We did not perform additional testing (e.g., repeat blood pressure measurements or ambulatory blood pressure recording) to verify normal blood pressure.

## Conclusion

Age- and sex trends of 4D flow CMR-derived flow component and KE parameters for both LV and RV were investigated in a sizable cohort. We found that for both LV and RV, flow components, average KEi_EDV_ for diastole and complete cardiac cycle neither change across age groups nor with age, whereas peak E-wave KEi_EDV_ decreases and peak A-wave KEi_EDV_ increases with age. Additionally, sex differences in 4D flow CMR-derived parameters were observed with women having reduced LV and RV residual volume and LV peak and average systolic KEi_EDV_, and increased RV direct flow and RV KEi_EDV_ of global, average diastole and peak E-wave. These findings may potentially explain the differences in individual responses of the heart to cardiopulmonary diseases and their treatment.

### Supplementary Information

**Additional file 1: Table S1**. Acquisition parameters of cine and 4D flow CMR imaging in two centres; **Table S2**. Flow components and kinetic energy (KE) parameters for the overall population, men and women; **Table S3**. Flow components and kinetic energy (KE) parameters according to age groups; **Table S4**. Right ventricular (RV) flow parameters between different scanners with age- and sex-matched subjects; **Table S5.** Impact of phase offset correction on the flow component and kinetic energy.

**Additional file 2: **Movies showing four-chamber views with right ventricle (RV) four flow components using particle tracing in a 29-year-old normal subject, a 36-year-old normal subject, a 49-year-old normal subject, a 55-year-old normal subject and a 64-year-old normal subject. Yellow circles denote the RV contours from stacks of short axis views. Color legend: green (RV direct flow), yellow (RV retained inflow), blue (RV delayed ejection flow), red (RV residual volume).

## Data Availability

The datasets used and/or analysed during the current study are available from the corresponding author on reasonable request.

## Abbreviations

1.5 T: 1.5 Telsa
3.0 T: 3.0 Tesla
2D: Two-dimensional
4D: Four-dimensional
AI: Artificial intelligence
BSA: Body surface area
CMR: Cardiovascular magnetic resonance
CPET: Cardiopulmonary exercise testing
ECG: Electrocardiogram
EDV: End-diastolic volume
EF: Ejection fraction
EPI: Echo-planar imaging
ES: End systole
ESV: End-systolic volume
GLS: Global longitudinal strain
IQR: Interquartile range
KE: Kinetic energy
KEi_EDV_: KE normalized to EDV
LV: Left ventricle
LVEDV: Left ventricular end-diastolic volume
LVEF: Left ventricular ejection fraction
LVESV: Left ventricular end-systolic volume
PAH: Pulmonary arterial hypertension
PC: Phase-contrast
rTOF: Repaired tetralogy of Fallot
RA: Right atrial
RV: Right ventricle
RVEDV: Right ventricular end-diastolic volume
RVEF: Right ventricular ejection fraction
RVESV: Right ventricular end-systolic volume
SD: Standard deviation
SENSE: Sensitivity encoding
SV: Stroke volume
TA: Tricuspid annular
