# Supplementary material for: Age- and sex-specific reference values of biventricular flow components and kinetic energy by 4D flow cardiovascular magnetic resonance in healthy subjects
Source: J Cardiovasc Magn Reson. 2023 Sep 18;25:50. doi: 10.1186/s12968-023-00960-x (PMC10506211; doi:10.1186/s12968-023-00960-x)
Supplement: Supplementary file 1 — Additional file 1: Table S1. Acquisition parameters of cine and 4D flow CMR imaging in two centres; Table S2. Flow components and kinetic energy (KE) parameters for the overall population, men and women; Table S3. Flow components and kinetic energy (KE) parameters according to age groups; Table S4. Right ventricular (RV) flow parameters between different scanners with age- and sex-matched subjects; Table S5. Impact of phase offset correction on the flow component and kinetic energy. [file 12968_2023_960_MOESM1_ESM.docx]

**Table S1.** Acquisition parameters of cine and 4D flow CMR imaging in two centres.

| Vendor | Philips | Siemens |
| --- | --- | --- |
| Magnetic field strength | 3.0T | 1.5T |
| Pulse sequence | Spoiled gradient echo | Spoiled gradient echo |
| **Cine images** | | |
| TR/TE (ms) | 2.8/1.4 | 3.4/1.3 |
| Flip angle (°) | 45 | 72 |
| Field of view (mm^2^) | 320 x 320 | 320 x 260 |
| Slice thickness, mm | 8 | 8 |
| Cardiac phases | 30 | 30 |
| **4D flow images** | | |
| Acceleration method | EPI factor 5 and SENSE factor 2 | GRAPPA factor 2 |
| Field of view (mm^2^) | 340 × 340 | 340 × 236.8 |
| Slice orientation | Coronal | Sagittal |
| Acquired voxel size (mm^3^) | 3.0 × 3.0 × 3.0 | 3.0 × 3.0 × 3.0 |
| Bandwidth, Hz/pixel | 1419 | 495 |
| TR/TE (ms) | 6.4-12.0/3.3-4.0 | 8-12/2.94 |
| Flip angle (°) | 10 | 9 |
| Cardiac phases | 30 | 23 (20, 24)* |
| VENC (cm/s) | 150 - 220 | 150 - 220 |
| Cardiac gating | Retrospective ECG gating | Prospective ECG gating |
| Respiratory motion | Free breathing | Free breathing |
| Scan time (min) | 5 - 10 | 20 |

*4D* four-dimensional, *CMR* cardiovascular magnetic resonance, *ECG* electrocardiogram, *EPI* echo-planar imaging, *GRAPPA* generalized autocalibrating partially parallel acquisition, *SENSE* sensitivity encoding, *TR* repetition time, *TE* echo time, *VENC* velocity encoding. *Median (25^th^ percentile, 75^th^ percentile).

**Table S2.** Flow components and kinetic energy (KE) parameters for the overall population, men and women

|  | **All**  **(n = 163)** | **Men**  **(n = 95)** | **Women**  **(n = 68)** | ***P*** |
| --- | --- | --- | --- | --- |
| **LV flow components** | | | | |
| Direct flow, % | 34 ± 7 | 33 ± 7 | 35 ± 6 | 0.080 |
| Retained inflow, % | 15 ± 5 | 15 ± 5 | 16 ± 4 | 0.135 |
| Delayed ejection flow, % | 17 ± 5 | 17 ± 5 | 18 ± 5 | 0.106 |
| Residual volume, % | 33 ± 7 | 35 ± 7 | 31 ± 6 | **<0.001** |
| **LV KEi_EDV_ parameters** | | | | |
| Global KEi_EDV_, μJ/ml | 9.9 ± 3.6 | 9.9 ± 3.6 | 9.9 ± 3.5 | 0.973 |
| Peak systolic KEi_EDV_, μJ/ml | 20.2 ± 8.5 | 21.4 ± 8.0 | 18.6 ± 9.0 | 0.043 |
| Average systolic KEi_EDV_, μJ/ml | 11.0 ± 4.3 | 11.5 ± 4.0 | 10.2 ± 4.7 | 0.062 |
| Average diastolic KEi_EDV_, μJ/ml | 9.4 ± 3.6 | 9.1 ± 3.6 | 9.8 ± 3.5 | 0.183 |
| Peak E-wave KEi_EDV_, μJ/ml | 26.0 ± 9.5 | 24.6 ± 9.6 | 28.0 ± 9.0 | 0.022 |
| Peak A-wave KEi_EDV_, μJ/ml | 12.6 ± 7.8 | 12.8 ± 7.8 | 12.2 ± 7.8 | 0.626 |
| KEi_EDV_ E/A ratio | 2.95 ± 2.34 | 2.82 ± 2.50 | 3.15 ± 2.09 | 0.374 |
| **RV flow components** |  |  |  |  |
| Direct flow, % | 36 ± 7 | 35 ± 7 | 37 ± 7 | 0.014 |
| Retained inflow, % | 17 ± 5 | 17 ± 5 | 17 ± 5 | 0.587 |
| Delayed ejection flow, % | 17 ± 5 | 15 ± 4 | 17 ± 5 | 0.019 |
| Residual volume, % | 31 ± 7 | 33 ± 7 | 29 ± 7 | **0.001** |
| **RV KEi_EDV_ parameters** |  |  |  |  |
| Global KEi_EDV_, μJ/ml | 8.8 ± 2.9 | 8.4 ± 2.9 | 9.3 ± 2.8 | 0.032 |
| Peak systolic KEi_EDV_, μJ/ml | 22.6 ± 7.5 | 22.9 ± 8.0 | 22.2 ± 6.7 | 0.554 |
| Average systolic KEi_EDV_, μJ/ml | 12.3 ± 3.9 | 12.4 ± 4.2 | 12.3 ± 3.5 | 0.952 |
| Average diastolic KEi_EDV_, μJ/ml | 6.6 ± 2.7 | 6.2 ± 2.6 | 7.3 ± 2.8 | **0.007** |
| Peak E-wave KEi_EDV_, μJ/ml | 13.4 ± 5.2 | 12.3 ± 5.1 | 15.0 ± 5.0 | **0.001** |
| Peak A-wave KEi_EDV_, μJ/ml | 9.2 ± 4.7 | 9.0 ± 4.3 | 9.5 ± 5.3 | 0.506 |
| KEi_EDV_ E/A ratio | 1.80 ± 1.19 | 1.65 ± 1.02 | 2.03 ± 1.37 | 0.048 |

Data were represented as mean ± SD. *EDV* end-diastolic volume, *KEi_EDV_* KE normalized to EDV, *LV* left ventricle, *RV* right ventricle. Bonferroni significance levels for LV flow parameters, LV KEi_EDV_ parameters, RV flow parameters and RV KEi_EDV_ parameters are calculated as 0.05/4 = 0.0125; 0.05/7 = 0.007; 0.05/4 = 0.0125 and 0.05/7 = 0.007, respectively.

**Table S3.** Flow components and kinetic energy (KE) parameters according to age groups

|  | **20-29**  **(n = 31)** | **30-39**  **(n = 45)** | **40-49**  **(n = 38)** | **50-59**  **(n = 29)** | **60-70**  **(n = 20)** | ***P^1^*** |
| --- | --- | --- | --- | --- | --- | --- |
| **LV flow components** | | | | | | |
| Direct flow, % | 37 ± 6 | 33 ± 6 | 33 ± 7 | 33 ± 6 | 32 ± 8 | 0.090 |
| Retained inflow, % | 14 ± 4 | 16 ± 5 | 15 ± 4 | 16 ± 4 | 16 ± 6 | 0.256 |
| Delayed ejection flow, % | 16 ± 4 | 18 ± 6 | 17 ± 5 | 17 ± 7 | 18 ± 5 | 0.714 |
| Residual volume, % | 33 ± 7 | 33 ± 6 | 33 ± 7 | 33 ± 6 | 33 ± 7 | 0.997 |
| **LV KEi_EDV_ parameters** | | | | | | |
| Global KEi_EDV_, μJ/ml | 9.9 ± 2.8 | 9.8 ± 3.7 | 9.7 ± 3.3 | 10.2 ± 4.7 | 10.2 ± 3.2 | 0.978 |
| Peak systolic KEi_EDV_, μJ/ml | 19.3 ± 6.7 | 20.9 ± 7.8 | 20.0 ± 7.9 | 21.0 ± 12.9 | 19.5 ± 6.6 | 0.906 |
| Average systolic KEi_EDV_, μJ/ml | 10.3 ± 2.9 | 11.0 ± 4.3 | 10.8 ± 3.7 | 11.5 ± 6.7 | 11.5 ± 3.2 | 0.843 |
| Average diastolic KEi_EDV_, μJ/ml | 9.7 ± 3.1 | 9.3 ± 3.8 | 9.1 ± 3.3 | 9.5 ± 4.3 | 9.4 ± 3.6 | 0.969 |
| Peak E-wave KEi_EDV_, μJ/ml | 31.0 ± 10.2 | 28.3 ± 8.6 | 25.0 ± 7.5 | 21.9 ± 9.9*^$^ | 21.1 ± 8.5*^$^ | **<0.001** |
| Peak A-wave KEi_EDV_, μJ/ml | 9.0 ± 3.5 | 10.2 ± 7.6 | 11.9 ± 6.0 | 16.4 ± 9.4*^$^ | 18.7 ± 8.0*^$#^ | **<0.001** |
| KEi_EDV_ E/A ratio | 3.72 ± 1.50 | 4.31 ± 3.30 | 2.62 ± 1.57^$^ | 1.63 ± 0.89*^$^ | 1.33 ± 0.81*^$^ | **<0.001** |
| **RV flow components** | | | | | | |
| Direct flow, % | 36 ± 7 | 35 ± 7 | 35 ± 7 | 37 ± 8 | 37 ± 5 | 0.321 |
| Retained inflow, % | 16 ± 3 | 17 ± 6 | 17 ± 4 | 18 ± 6 | 18 ± 5 | 0.609 |
| Delayed ejection flow, % | 17 ± 4 | 16 ± 4 | 16 ± 5 | 15 ± 5 | 15 ± 6 | 0.342 |
| Residual volume, % | 33 ± 7 | 32 ± 7 | 30 ± 8 | 31 ± 8 | 29 ± 8 | 0.435 |
| **RV KEi_EDV_ parameters** | | | | | | |
| Global KEi_EDV_, μJ/ml | 9.2 ± 2.4 | 8.8 ± 2.6 | 8.9 ± 2.6 | 9.0 ± 4.0 | 7.7 ± 2.7 | 0.476 |
| Peak systolic KEi_EDV_, μJ/ml | 24.0 ± 7.8 | 23.3 ± 7.5 | 23.0 ± 7.7 | 21.4 ± 7.1 | 20.1 ± 6.9 | 0.323 |
| Average systolic KEi_EDV_, μJ/ml | 13.4 ± 3.9 | 12.3 ± 3.7 | 12.6 ± 4.0 | 11.7 ± 4.4 | 11.0 ± 3.6 | 0.258 |
| Average diastolic KEi_EDV_, μJ/ml | 6.8 ± 1.9 | 6.7 ± 2.6 | 6.6 ± 2.2 | 7.1 ± 4.1 | 5.9 ± 2.4 | 0.646 |
| Peak E-wave KEi_EDV_, μJ/ml | 15.4 ± 5.6 | 14.4 ± 4.8 | 13.1 ± 4.1 | 12.6 ± 6.3 | 9.9 ± 3.9*^$^ | **0.002** |
| Peak A-wave KEi_EDV_, μJ/ml | 7.4 ± 3.0 | 8.1 ± 4.9 | 9.1 ± 3.5 | 12.1 ± 6.4*^$^ | 10.4 ± 4.0 | **<0.001** |
| KEi_EDV_ E/A ratio | 2.37 ± 1.36 | 2.36 ± 1.40 | 1.64 ± 0.80^$^ | 1.16 ± 0.65*^$^ | 1.01 ± 0.38*^$^ | **<0.001** |

Data were represented as mean ± SD. *EDV* end-diastolic volume, *KEi_EDV_* KE normalized to EDV, *LV* left ventricle, *RV* right ventricle. ^1^*P* value from one-way ANOVA analysis with Bonferroni correction. *Significant difference compared with 20-29 age group; ^$^significant difference compared with 30-39 age group; ^#^significant difference compared with 40-49 age group.

**Table S4.** Right ventricular (RV) flow parameters between different scanners with age- and sex-matched subjects

|  | **Philips 3.0T with EPI (n=22)** | **Siemens 1.5T with non-EPI (n=22)** | ***P*** |
| --- | --- | --- | --- |
| Age, years | 43 ± 11 | 42 ± 11 | 0.926 |
| Gender, M/F | 10/12 | 10/12 | 1.000 |
| Percentage of valid particles, % | 90 ± 4 | 91 ± 8 | 0.530 |
| RV direct flow, % | 36 ± 7 | 36 ± 8 | 0.956 |
| RV retained inflow, % | 17 ± 4 | 17 ± 7 | 0.825 |
| RV delayed ejection flow, % | 17 ± 4 | 16 ± 6 | 0.277 |
| RV residual volume, % | 30 ± 8 | 31 ± 8 | 0.643 |
| RV retained inflow – RV delayed ejection flow, % | -0.1 ± 4.3 | 0.3 ± 7.6 | 0.842 |

Data were represented as mean ± SD. *EPI* echo-planar imaging.

**Table S5.** Impact of phase offset correction on the flow component and kinetic energy

|  | **Philips scanner (n=10)** | | | **Siemens scanner (n=10)** | | |
| --- | --- | --- | --- | --- | --- | --- |
|  | **With phase offset error correction** | **Without phase offset error correction** | ***P*** | **With phase offset error correction** | **Without phase offset error correction** | ***P*** |
| **LV flow components** | | | | | | |
| Direct flow, % | 34 ± 2 | 28 ± 5 | **0.011** | 33 ± 2 | 32 ± 3 | 0.631 |
| Retained inflow, % | 16 ± 3 | 16 ± 4 | 0.912 | 17 ± 4 | 19 ± 3 | 0.063 |
| Delayed ejection flow, % | 19 ± 2 | 21 ± 6 | 0.739 | 16 ± 2 | 17 ± 5 | 0.579 |
| Residual volume, % | 32 ± 4 | 35 ± 5 | 0.105 | 35 ± 4 | 31± 4 | 0.089 |
| **LV KEi_EDV_ parameters** | | | | | | |
| Global KEi_EDV_, μJ/ml | 7.8 ± 1.5 | 8.5 ± 1.9 | 0.436 | 8.7 ± 1.6 | 8.9 ± 2.0 | 0.853 |
| Peak systolic KEi_EDV_, μJ/ml | 16.0 ± 4.0 | 19.4 ± 5.7 | 0.190 | 20.3 ± 5.7 | 20.6 ± 6.3 | 0.971 |
| Average systolic KEi_EDV_, μJ/ml | 9.1 ± 2.6 | 10.5 ± 3.0 | 0.218 | 10.1 ± 2.3 | 10.2 ± 2.5 | 0.739 |
| Average diastolic KEi_EDV_, μJ/ml | 7.1 ± 1.2 | 7.5 ± 1.8 | 0.796 | 7.9 ± 1.9 | 8.4 ± 2.2 | 0.529 |
| Peak E-wave KEi_EDV_, μJ/ml | 26.6 ± 5.0 | 27.7 ± 6.6 | 0.684 | 25.0 ± 10.0 | 26.4 ± 11.7 | 0.684 |
| Peak A-wave KEi_EDV_, μJ/ml | 8.1 ± 3.0 | 8.4 ± 3.0 | 0.796 | 7.5 ± 3.6 | 8.1 ± 3.7 | 0.798 |
| KEi_EDV_ E/A ratio | 3.77 ± 1.61 | 3.65 ± 1.20 | 0.971 | 3.66 ± 2.60 | 3.54 ± 2.55 | 0.878 |
| **RV flow components** | | | | | | |
| Direct flow, % | 34 ± 6 | 35 ± 6 | 0.247 | 35 ± 6 | 35 ± 4 | 0.912 |
| Retained inflow, % | 17 ± 3 | 18 ± 3 | 0.436 | 17 ± 2 | 18 ± 6 | 0.739 |
| Delayed ejection flow, % | 20 ± 4 | 16 ± 5 | 0.123 | 15 ± 4 | 11 ± 4 | 0.052 |
| Residual volume, % | 30 ± 7 | 31 ± 7 | 0.971 | 33 ± 6 | 36 ± 7 | 0.165 |
| RV KEiEDV parameters | | | | | | |
| Global KEi_EDV_, μJ/ml | 7.8 ± 1.5 | 7.6 ± 1.3 | 1.000 | 8.0 ± 1.6 | 8.3 ± 2.0 | 0.796 |
| Peak systolic KEi_EDV_, μJ/ml | 20.9 ± 5.1 | 20.1 ± 4.4 | 0.912 | 24.6 ± 6.9 | 26.1 ± 7.1 | 0.631 |
| Average systolic KEi_EDV_, μJ/ml | 11.8 ± 2.7 | 11.4 ± 2.6 | 0.971 | 12.1 ± 2.8 | 11.7 ± 4.0 | 1.000 |
| Average diastolic KEi_EDV_, μJ/ml | 5.7 ± 1.1 | 5.7 ± 1.0 | 0.971 | 5.5 ± 1.2 | 5.8 ± 1.5 | 0.853 |
| Peak E-wave KEi_EDV_, μJ/ml | 15.5 ± 4.3 | 15.3 ± 4.2 | 0.971 | 12.5 ± 4.0 | 12.3 ± 3.1 | 1.000 |
| Peak A-wave KEi_EDV_, μJ/ml | 6.3 ± 2.7 | 6.3 ± 2.7 | 0.971 | 6.6 ± 2.1 | 6.4 ± 2.1 | 0.902 |
| KEi_EDV_ E/A ratio | 2.87 ± 1.35 | 2.83 ± 1.35 | 0.853 | 1.81 ± 0.70 | 1.93 ± 0.79 | 0.902 |

*P* values are from nonparametric Wilcoxon Signed-Ranks Test.
