# Supplementary figures and images for: Age- and sex-specific reference values of biventricular flow components and kinetic energy by 4D flow cardiovascular magnetic resonance in healthy subjects
Source: J Cardiovasc Magn Reson. 2023 Sep 18;25:50. doi: 10.1186/s12968-023-00960-x (PMC10506211; doi:10.1186/s12968-023-00960-x)

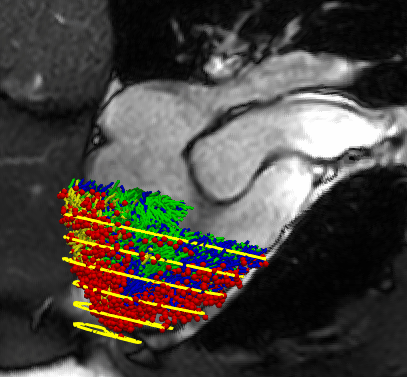

Supplement: Supplementary file 2 — Additional file 2: Movies showing four-chamber views with right ventricle (RV) four flow components using particle tracing in a 29-year-old normal subject, a 36-year-old normal subject, a 49-year-old normal subject, a 55-year-old normal subject and a 64-year-old normal subject. Yellow circles denote the RV contours from stacks of short axis views. Color legend: green (RV direct flow), yellow (RV retained inflow), blue (RV delayed ejection flow), red (RV residual volume). [file 12968_2023_960_MOESM2_ESM.zip › Additional file 2 - 29 year old.gif]

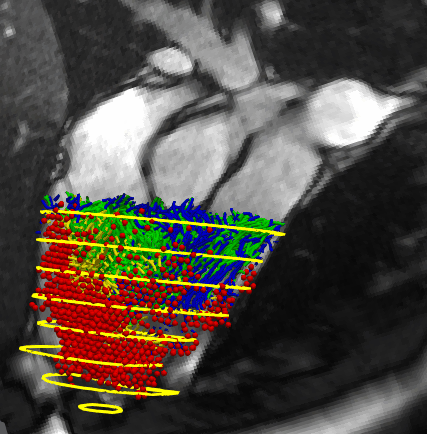

Supplement: Supplementary file 2 — Additional file 2: Movies showing four-chamber views with right ventricle (RV) four flow components using particle tracing in a 29-year-old normal subject, a 36-year-old normal subject, a 49-year-old normal subject, a 55-year-old normal subject and a 64-year-old normal subject. Yellow circles denote the RV contours from stacks of short axis views. Color legend: green (RV direct flow), yellow (RV retained inflow), blue (RV delayed ejection flow), red (RV residual volume). [file 12968_2023_960_MOESM2_ESM.zip › Additional file 2 - 36 year old.gif]

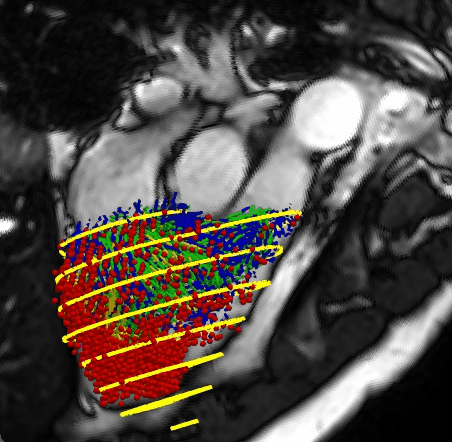

Supplement: Supplementary file 2 — Additional file 2: Movies showing four-chamber views with right ventricle (RV) four flow components using particle tracing in a 29-year-old normal subject, a 36-year-old normal subject, a 49-year-old normal subject, a 55-year-old normal subject and a 64-year-old normal subject. Yellow circles denote the RV contours from stacks of short axis views. Color legend: green (RV direct flow), yellow (RV retained inflow), blue (RV delayed ejection flow), red (RV residual volume). [file 12968_2023_960_MOESM2_ESM.zip › Additional file 2 - 49 year old.gif]

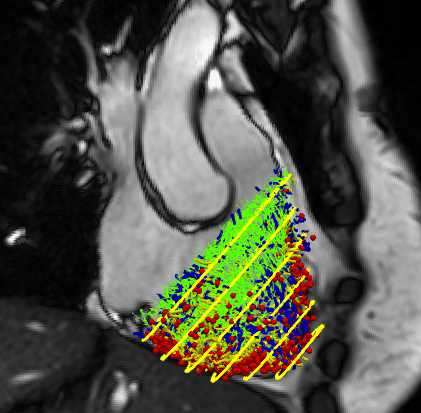

Supplement: Supplementary file 2 — Additional file 2: Movies showing four-chamber views with right ventricle (RV) four flow components using particle tracing in a 29-year-old normal subject, a 36-year-old normal subject, a 49-year-old normal subject, a 55-year-old normal subject and a 64-year-old normal subject. Yellow circles denote the RV contours from stacks of short axis views. Color legend: green (RV direct flow), yellow (RV retained inflow), blue (RV delayed ejection flow), red (RV residual volume). [file 12968_2023_960_MOESM2_ESM.zip › Additional file 2 - 55 year old.gif]

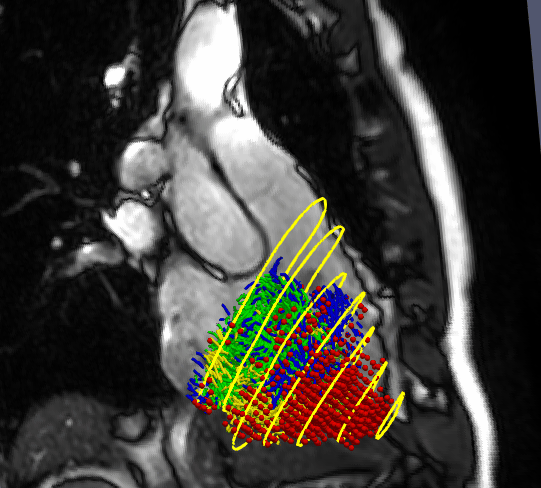

Supplement: Supplementary file 2 — Additional file 2: Movies showing four-chamber views with right ventricle (RV) four flow components using particle tracing in a 29-year-old normal subject, a 36-year-old normal subject, a 49-year-old normal subject, a 55-year-old normal subject and a 64-year-old normal subject. Yellow circles denote the RV contours from stacks of short axis views. Color legend: green (RV direct flow), yellow (RV retained inflow), blue (RV delayed ejection flow), red (RV residual volume). [file 12968_2023_960_MOESM2_ESM.zip › Additional file 2 - 64 year old.gif]
